# Successful transport across continents of GMP-manufactured and cryopreserved culture-expanded human fetal liver-derived mesenchymal stem cells for use in a clinical trial

**DOI:** 10.1016/j.reth.2024.06.012

**Published:** 2024-06-24

**Authors:** Ashis Kumar, Sowmya Ramesh, Lilian Walther-Jallow, Annika Goos, Vignesh Kumar, Åsa Ekblad, Vrisha Madhuri, Cecilia Götherström

**Affiliations:** a Department of Paediatric Orthopaedics, Christian Medical College, Vellore 632 004, Tamil Nadu, India; b Center for Stem Cell Research, a Unit of in Stem Bengaluru, Christian Medical College, Vellore 632 002, Tamil Nadu, India; c Division of Obstetrics and Gynaecology, Department of Clinical Science, Intervention and Technology, Karolinska Institutet, Stockholm, Sweden; d Sree Chitra Tirunal Institute for Medical Sciences & Technology, Thiruvananthapuram 695011, Kerala, India

**Keywords:** BOOST2B, Allogeneic stem cell therapy, Advanced therapy medicinal product, ATMP, Clinical trial, Bone differentiation

## Abstract

**Introduction:**

Cell therapy has been increasingly considered to treat diseases, but it has been proven difficult to manufacture the same product at multiple manufacturing sites. Thus, for a wider implementation an alternative is to have one manufacturing site with a wide distribution to clinical sites. To ensure administration of a good quality cell therapy product with maintained functional characteristics, several obstacles must be overcome, which includes for example transfer of knowledge, protocols and procedures, site assessment, transportation and preparation of the product.

**Methods:**

As the preparatory work for a clinical trial in India using fetal mesenchymal stem cells (fMSCs) developed and manufactured in Sweden, we performed a site assessment of the receiving clinical site, transferred methods, developed procedures and provided training of operators for handling of the cell therapy product. We further developed a Pharmacy Manual to cover the management of the product, from ordering it from the manufacturer, through transport, reconstitution, testing and administration at the clinical site. Lastly, the effect of long-distance transport on survival and function of, as well as the correct handling of the cell therapy product, was evaluated according to the pre-determined and approved Product Specification.

**Results:**

Four batches of cryopreserved human fetal liver-derived fMSCs manufactured according to Good Manufacturing Practice and tested according to predetermined release criteria in Sweden, were certified and transported in a dry shipper at −150 °C to the clinical site in India. The transport was temperature monitored and took three–seven days to complete. The thawed and reconstituted cells showed more than 80% viability up to 3 h post-thawing, the cell recovery was more than 94%, the cells displayed the same surface protein expression pattern, differentiated into bone, had stable chromosomes and were sterile, which conformed with the data from the manufacturing site in Sweden.

**Conclusions:**

Our study shows the feasibility of transferring necessary knowledge and technology to be able to carry out a clinical trial with a cell therapy product in distant country. It also shows that it is possible to transport a cryopreserved cell therapy product over long distances and borders with retained quality. This extends the use of cryopreserved cell therapy products in the future.

## Highlights

• Transfer of specialised protocols, methods and knowledge between distant sites.
• Transport of an advanced cell therapy product over continents.
• Verification of retained quality and efficacy of an advanced cell therapy product.
• Processes used in cell therapy clinical trials are generally not widely published.

## 1 Introduction

More recently, with increasing globalization and success in various clinical trials, the field of regenerative medicine has expanded to include translation of a wide range of advanced therapy medicinal products (ATMP) [[1], [2], [3]]. ATMPs are manufactured under the regulatory framework for Good Manufacturing Practice (GMP) [1,4], requiring that any manufactured product must be validated according to set criteria and fulfil the pre-set Product Specification [5,6]. It might be a challenging and time-consuming task to align the production between manufacturing sites to fulfil regulatory requirements and also to ensure that data will be comparable within the same clinical trial [7,8]. A preferable approach may instead be to manufacture the cells at one site and establish functional procedures for transport, transfer of knowledge and protocols and training for handling of the product at the receiving clinical sites. This approach may prove difficult for autologous products, where the starting material is e.g., blood or tissue harvested from the patient to be treated, and with products with a short stability after production where cryo-preservation is not an option. However, several emerging cell therapies such as allogenic mesenchymal stem cells (MSCs) for treatment of various diseases and NK-cells and CAR T-cells for treatment of various cancers and adoptive immune cell therapy, are good candidates for an off-the shelf approach, where cells can be stored long term as cryopreserved and sent to the clinical trial site upon request [[9], [10], [11]].

The latter is a rather straight forward scenario when using ATMPs that can be thawed and administered bedside. However, some ATMPs need a reconstitution step before administration. In this case it is important that the clinical trial site has access to adequate localities and equipment, as well as operators who are well trained in performing the reconstitution and handling of the product, in order to retain the quality [12]. The cryopreserved cell therapy product should survive, remain viable and sterile and retain their phenotype without compromising their potency after thawing and reconstitution.

MSCs have previously been reported to be safe and efficient when treating a small group of patients with severe types of Osteogenesis Imperfecta (OI) [[13], [14], [15], [16]].

The principal theory regarding the Mechanism of Action for fMSCs in treating OI centres on the integration of donor cells within the patient's extracellular matrix, leading to the synthesis of collagen and the formation of healthy bone. Additionally, the Mechanism of Action is believed to encompass secondary mechanisms such as paracrine signalling, enhanced angiogenesis, and stimulation or strengthening of the patient's endogenous cells and extracellular matrix. At the current phase I/II stage of this cell therapy's development, assessing the *in vitro* osteogenic differentiation capacity serves as a reliable metric for evaluating the efficacy of the fMSCs. Establishing more definitive correlations between the *in vitro* results and clinical outcomes is a goal set for subsequent phases of the development.

In preparation for the registered and approved clinical trial Boost to Brittle Bones (BOOST2B, ClinicalTrials.gov Identifier: NCT04623606), cryopreserved fMSCs were transported from the manufacturing site in Sweden to the clinical site in India for use in a clinical trial on children with severe type of OI. The same cell product is investigated in a parallel trial in Europe; BOOSTB4 (ClinicalTrials.gov Identifier: NCT03706482).

To align expectations and ensure high quality when initiating a project with many stakeholders involved, it is crucial to define project objectives and deliverables, involve all stakeholders in the planning phase, regularly review and update project progress, and establish clear communication channels, throughout the project lifecycle. During a brainstorming session with all stakeholders from both countries present, including laboratory technicians, doctors, nurses, pharmacists, quality- and regulatory experts and project coordinators, we identified areas that were considered critical for a successful project performance, which included:

- Transfer of protocols, methods and training of staff at the clinical site, including a site assessment to verify adequate facilities and equipment,
- Transport of the cryopreserved cell therapy product from Sweden to India including export and import permissions from the respective countries,
- Storage and handling of the cryopreserved cell therapy product at the clinical site including adequate equipment and temperature control,
- Thawing and reconstitution of the cell therapy product and testing for retained quality of the reconstituted cells,
- Delivery of the cell therapy product to the clinic after reconstitution where a Pharmacy Manual was developed for consistent handling.

In this study, we describe these different steps to ensure a safe, efficient and streamlined handling of the cell therapy product at the clinical site, with the aim to demonstrate that a cell therapy product can indeed be manufactured at one site and used at another, without compromising product quality, even when long transportation and a reconstitution step is required.

## 2 Material and methods

### 2.1 Ethics and research approval

The BOOST2B trial has approval from the local institutional review board at Christian Medical College (CMC), Vellore (IRB Min no. 10384) and the Central Drug Standard Control Organisation, the Directorate General of Health Services, the Ministry of Health & Family Welfare, Government of India (F.No.Stm-CI/15/CMC/18-BD) and the Health Ministry's (Indian Council of Medical Research, ICMR) Screening Committee (HMSC 2019-7846) for import of the fMSCs. The trial is registered at the National Clinical Trial Registry (REF/2017/06/014491) and at ClinicalTrials.gov (NCT04623606). The procurement and manufacturing are approved by the Ethical Review Authority in Sweden (2018/1732-31) and the National Board of Health and Welfare (8.1-28985/2018), and was performed according to GMP at Karolinska University Hospital (KUH) in Sweden. Informed oral and written consent was obtained from all women donating the tissue. The trial is carried out according to Good Clinical Practice (GCP), and according to applicable national laws and regulations as stated in the Declaration of Helsinki.

### 2.2 Study design and set-up

**Fig. 1:**
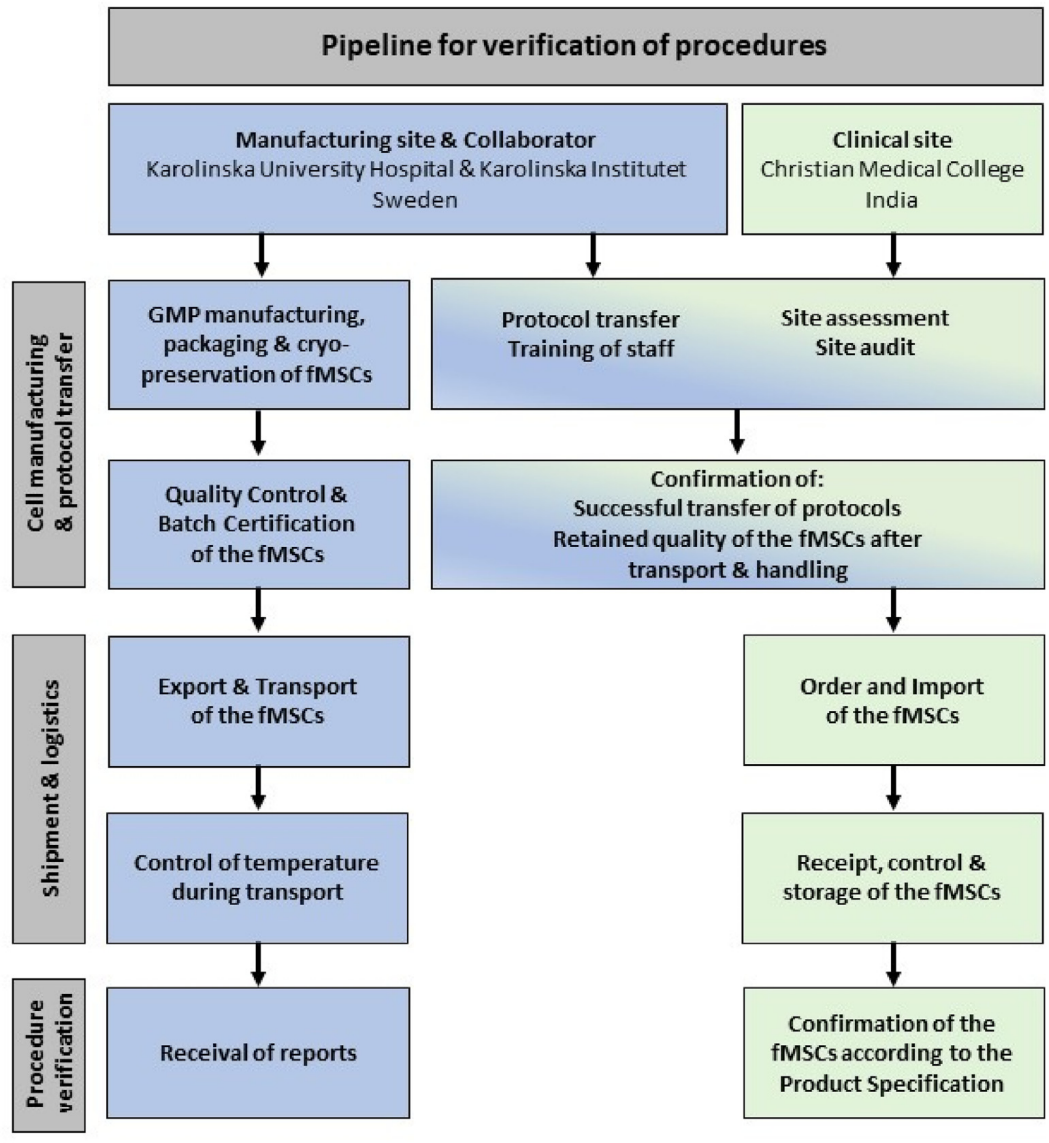
**Pipeline displaying the procedures involved in the project.** Pipeline for verification of the procedures involved in the protocol and knowledge transfer and transport of clinical grade cryopreserved fMSCs for clinical use across continents from Sweden to India.

This study was performed to prepare for the BOOST2B trial at CMC in Vellore, India using fMSCs manufactured and batch certified by the GMP manufacturing site KUH and Karolinska Institutet (KI) in Sweden. Both sites have established GMP facilities and have a long experience in developing and performing clinical trials with cell therapy products. Protocol transfer was performed to the clinical site, including training of operators in methods specific for the cell therapy product in question, and a site assessment was carried out. The cells were thereafter exported, imported and transported to the clinical site in India. Upon arrival, the cells were stored as cryopreserved until further testing and use. Lastly, since the fMSCs require a reconstitution step before use in patients, verification of retained quality was performed after thawing, reconstitution and transport to and handling at the clinical site. See Fig. 1 for a schematic pipeline view of the study.

### 2.3 Manufacture and cryopreservation of the cell therapy product at the manufacturing site

The fMSCs were isolated from liver tissue from legal terminations in the first trimester. Informed oral and written consent was obtained from all women donating the tissue (see above for information on ethical permits). The fMSCs were manufactured and tested according to the predetermined and approved release criteria at the GMP facility at KUH in Sweden. After isolation, the cells were expanded in a two-step procedure and were then detached from the cell culture flasks, washed, and cryopreserved in pre-cooled physiological NaCl (80%, v/v, B. Braun Melsungen AG, Melsungen, Germany), Human Serum Albumin (HSA, 10%, v/v, Baxalta, Takeda Pharmaceutical Company Limited Osaka, Japan) and dimethylsulphoxide (DMSO) (10%, v/v, WAK Chemie Medical GmbH, Steinbach, Germany). One or three mL of a solution consisting of 5 × 10^6^ cells/mL were placed in 2 or 5 mL cryovials with external screw cap without a gasket (TPP Techno Plastic Products AG, Trasadingen, Switzerland). The vials were labelled and placed in a Cool cell (Corning, NY, USA) in −80 °C for 24 h and thereafter moved to temperature-controlled freezers at −150 °C for long-term storage until transport. Long-term as well as in-use stability had been determined previously as part of the stability program for the fMSCs at the manufacturer in Sweden. Long term storage is currently ≥36 months for the cryopreserved fMSCs, and the in-use stability i.e., the stability of the product from the time of thawing, during reconstitution and administration to the patient has been determined to ≥90 min.

From one donor 10–13 batches can be manufactured, and each batch is enough to treat 2–3 patients with 4 doses each. In this study 4 batches derived from 2 donors were used. Three of the batches (derived from one single donor) were approved for clinical use and cells from these are also used in the BOOST2B clinical trial. The fourth batch used in this study (from another donor) was research grade, meaning that it was not intended for use in the clinical trial, and was merely utilized to verify procedures in this present study.

### 2.4 Certification of the cell therapy product at the manufacturing site

The manufacturer provides a proof of certification of the cell therapy product. It contain the quality control data according to the specification of the final product (release criteria) and the starting material specification and includes: viability, recovery, number of population doublings, population doubling time, cell surface markers, bone differentiation, karyotype, analysis of mutations associated to skeletal dysplasia, virus and bacteria screening (HIV, Hepatitis A, B, C and E, Syphilis, Human T-lymphotropic virus type 1 and 2, Parvovirus, Zika virus, West Nile virus, Cytomegalovirus, Chlamydia and Gonorrhoea) and tests for mycoplasma, sterility and endotoxin (see Table 1 for the methods along with their release criteria).

**Table 1:** Summary of methods, acceptance criteria and results from the analyses of the fMSCs at the manufacturing site in Sweden and the clinical site in India.

Test	Method	Acceptance criteria	Results Manufacturing site (n = 4)	Results Clinical site (n = 4)
Viability at thawing^a^	Cell count^b^	≥70%	92.3% (89.2–97.0%)	90.0% (86.0–93.0%)
Recovery at thawing	Cell count^b^	≥3.5 × 10^6^ viable cells/mL/≥70%	4.6 × 10^6^/92.0% (4.5–4.9 × 10^6^/90.0–98.0%)	4.5 × 10^6^/90% (4.4–4.6 × 10^6^/88–92%)
Identity (% viable fMSCs)^a^	Flow cytometry^b^	≥85% for CD73 & CD90	97.5% (90.9–99.6%)	97.9% (97.2–98.4%)
Purity (% contaminating cells)^a^	Flow cytometry^b^	≤5% for CD31 ≤5% for CD45 ≤5% for HLA class II	0.2% (0.0–1.0%) 0.3% (0.0–1.1%) 1.2% (0.1–2.0%)	0.6% (0.4–0.8%) 0.9% (0.4–2.5%) 1.2% (0.2–3.1%)
Potency assay: Bone differentiation	Bone differentiation assay (*in vitro*)^b^	≥2.5-fold differentiation over negative control	13.3-fold (6.3–22.5)	5.7-fold (4.2–7.0)
Chromosome stability	Cytogenetics	46, XX or 46, XY^c^	46, XX	46, XX
Sterility	Sweden: Ph.Eur. 2.6.1 India: Microbiology^b^	Sterile	Sterile	Sterile
Mycoplasma	Sweden: Ph.Eur. 2.6.14 India: PCR^b^	Negative, no mycoplasma detected	Negative	Negative
Endotoxin	Sweden: Ph.Eur. 2.6.7 (qPCR) India: Endosafe^b^	≤0.5 EU/mL	<0.5 EU/mL	<0.5 EU/mL
Appearance of reconstituted fMSCs	Visual inspection	Colourless cell suspension, free of visible particulate matter	Pass	Pass

Ph.Eur. = European Pharmacopoeia.

a Values expressed as mean (range).

b In-house protocol.

c ≥ 2 identical clonal numerical abnormal metaphases on 20–25 metaphases is not accepted.

### 2.5 Protocol and knowledge transfer and assessment of the clinical site

Protocols and methods needed for the thawing, reconstitution, re-testing of the fMSCs were transferred to the clinical site (Table 1). Operators were trained in all procedures, firstly during a visit to the manufacturing site in Sweden, and later on-site at the GMP facility at the clinical site in India.

A Pharmacy Manual for the clinical site was developed to describe the handling of the fMSCs. A Pharmacy Manual is a useful tool to ensure consistency on management at clinical trial sites. Key components of a Pharmacy Manual are e.g. information on the drug product such as composition, dosing, storage and stability (long-term and in-use), supply processes, dose administration (including instructions for reconstitution when relevant) and drug accountability. For the BOOST2B trial, as is the custom for such documents, we developed the Pharmacy Manual in close collaboration between the manufacturing- and the clinical sites, to tailor it to the specific requirements involved in receiving and preparing the fMSCs for dosing of the participants in the BOOST2B trial. The BOOST2B Pharmacy Manual describes in detail the procedures and responsibilities for ordering, shipment, receipt, storage and stability, release, reconstitution, labelling, transport, administration and accountability of the cell therapy product in the clinical trial. Appendices include Template for labels, syringes and outer transportation bag, form for Order and delivery of cryopreserved cell therapy product, form for Order of reconstituted cell therapy product, a Dose reconstitution protocol, a working protocol for the Administration of reconstituted cell therapy product, a Dose release form, an Infusion protocol, a worksheet for recording the Medical Devices & Components, a Drug accountability sheet, and a Line clearance protocol. Included is also a Worksheet for recording of body weight, a Subject log, a Temperature log, a Delegation log and the curriculum vitae of the Indian Principal Investigator for the trial.

A site assessment was performed, which included GMP certification, storage conditions and temperature monitoring, localities, equipment for the reconstitution, qualification of the personnel and written routines for documentation and reporting. A site visit at the clinical site by the manufacturer was also performed as well as training of the operators at the clinical site to perform e.g. the confirmatory assays and the reconstitution.

### 2.6 Transport, receival and storage of the fMSCs at the clinical site

**Fig. 2:**
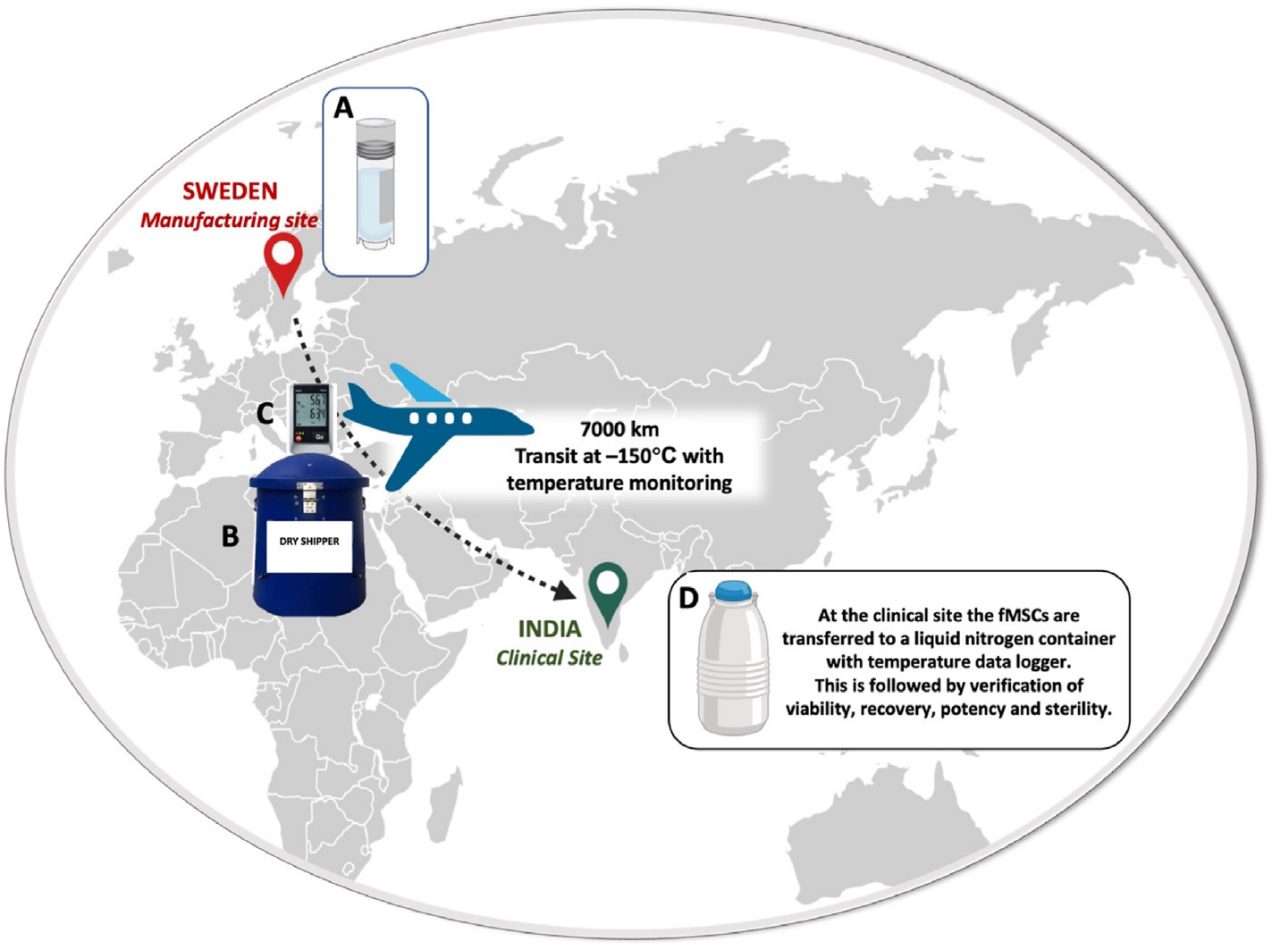
**Graphical figure showing the transport of the fMSCs from Sweden to India.** The fMSCs **(A)** were at the manufacturing site in Sweden packed according to regulations and transported to India in a dry shipper **(B)** with a temperature logger **(C)** using a courier. Upon arrival at the clinical site in India the cells were controlled and transferred to a liquid nitrogen container **(D)** equipped with a temperature logger, for long-term storage.

Necessary approvals for export and import of biological material were obtained by Swedish and Indian authorities. To import drugs of human origin for pharmaceutical purposes to India, the drug must comply with regulations overseen by India's Central Drugs Standard Control Organization. From the sender the following documents are needed; an air waybill, a commercial invoice, a packing list, a Material Safety Data Sheet (MSDS) – with details of any chemical substances, their potential hazards and relevant handling instructions. The MSDS is also required for any chemicals used to preserve biologics, as they may be classified as dangerous goods. The receiver needs to provide KYC (Know Your Customer) documentation (for first shipment with the carrier) and an import licence from the ICMR for pharmaceutical products. After ordering the cells from the manufacturing site in Sweden, vials of certified fMSCs (n = 4, all XX) were transported to India with sufficient cells for all four planned doses per participant (Fig. 2). The transport was performed in the vapour phase of liquid nitrogen in a dry shipper provided by the courier at less than −150 °C via air carriage and ground freight. The dry shipper can keep a temperature of at least −150 °C over 12 days, as has been validated by the courier. The temperature had to be lower than −130 °C throughout the transport. A data logger was used to monitor the temperature during the transport, which was controlled by the clinical site after completed transport, and thereafter reported to the manufacturing site. Each transport was accompanied by a batch certificate from the manufacturer. Upon receipt, the vials were counted, the identity and integrity of the vials confirmed, and the vials were immediately transferred to a liquid nitrogen tank at −196 °C. Monitoring of the temperature was performed according to standard operating procedures at the clinical site using a digital data logger (Tempmate GmbH, Heilbronn, Germany).

### 2.7 Verification of retained quality of the fMSCs at the clinical site after transport and handling

Aliquots (one test vial) from the four certified cell-batches were tested upon arrival at the clinical site in India to assess retained purity, identity and efficacy after the transport and handling at the clinical site. The tests performed are listed in the Product Specification along with the methods and their acceptance criteria (Table 1), and are described in more detail below. This confirmatory testing was requested by the local institutional review board at CMC in Vellore, India. All reagents, consumables, syringes and equipment were obtained either from the same company with the same catalogue number as used by the manufacturer or validated for safe use at the clinical site.

#### 2.7.1 Transfer of methods and details of diverse methods used at the two sites

The in-house methods cell recovery and viability, flow cytometry for immunophenotyping, bone differentiation and karyotyping were transferred from the manufacturing site to the clinical site. The methods for detection of sterility, and for presence of any mycoplasma and endotoxins differed between the sites. These methods comply with the regulations in India and are approved by the Central Drugs Standard Control Organization in India for use in the BOOOST2B trial.

#### 2.7.2 Cell recovery and viability

Cryopreserved fMSCs (5 × 10^6^ per mL, 1 mL/vial) were thawed in a 37 °C water bath until a small crystal of ice remained in the vial. One mL of room tempered (25 °C) 0.9% NaCl (Fresenius Kabi, Bad Homburg, Germany) + 8% Human Serum Albumin (HSA, Baxter, IL, USA) was added to the cell suspension and mixed gently. The cells were transferred to a 50-mL tube and 48 mL NaCl with 8% HSA was added and the cells were centrifuged at 500 *g* for 7 min at room temperature in a swing-out rotor centrifuge. The supernatant was removed, and the pellet was re-suspended in 1 mL 0.9% NaCl with 8% HSA, and cell counting was performed by the Trypan blue (Gibco™, MA, USA) dye exclusion assay using a manual Neubauer counting chamber (Paul Marienfeld GmbH, Germany). Both live and dead cells were counted. To pass the release criteria, the cell viability after thawing must be >70%, and the cell recovery must be >3.5 × 10^6^ viable fMSCs/mL (70%), and the suspension must be homogeneous with no aggregates (Table 1). For the purpose of this study, we expected the results to be within 10% of the values disclosed by the manufacturer.

#### 2.7.3 Immunophenotyping

The fMSCs were thawed as described above, diluted in 5 mL incubation buffer consisting of phosphate buffer saline (PBS, Sigma-Aldrich, MO, USA) and 2% fetal bovine serum (FBS, Gibco™, MA, USA), centrifuged, and resuspended in incubation buffer. For each batch of fMSCs, 50,000 cells per tube were added and stained with the antibodies (Table 2), followed by an incubation of 20 min at room temperature in the dark. After staining, the fMSCs were washed with incubation buffer, centrifuged, and re-suspended in 250 μl incubation buffer. A minimum of 15,000 events were acquired using ARIAIII cytometer (BD Biosciences, NJ, USA), and analysis of the data was performed using CytExpert software (v2.5 Beckman Coulter, IN, USA). Unstained fMSCs were used for determining the gating strategy.

**Table 2:** List of antibodies.

Antigen and clone	Conjugate	Volume used/assay	Manufacturer
CD90, Clone 5E10	FITC	6 μL	BD Bioscience, NJ, USA
CD73, Clone AD2	APC	5 μL	BD Bioscience, NJ, USA
CD45, Clone 2D1	PerCP	5 μL	BD Bioscience, NJ, USA
CD31, Clone L133.1	PE	5 μL	BD Bioscience, NJ, USA
HLA class II (DR, DP, DQ) Clone Tu39	PE-Cy7	4 μL	BD Bioscience, NJ, USA

#### 2.7.4 Bone differentiation

Thawed fMSCs (see above) were plated at a density of 3.5 × 10^3^ cells/cm^2^ in a 12 well plate (CellBind, Corning, AZ, USA) in fMSC culture media; Dulbecco's modified Eagle Medium – Low Glucose (DMEM-LG, Life Technologies Ltd, Paisley, Scotland, UK) supplemented with 10% FBS and 1% antibiotics/antimycotics (Anti-Anti, Life Technologies Ltd) at 37 °C with 5% CO_2_. After 24 h, either an osteogenic differentiation media (StemMACS, Miltenyi Biotec, NRW, Germany) or fMSC culture media was added. The media was changed every 3–4 days for 14–21 days after which Alizarin Red S staining was performed for quantification of osteogenic differentiation. The cells were washed very gently 2 times with 1 mL PBS/well, and then fixed with 1 mL of 4% formalin (Fisher Scientific, NH, USA) per well for 1 h and washed twice with 1 mL distilled water (dH2O)/well. The cells were then stained with 1 mL freshly made 40 mM Alizarin Red S, pH 4.2 (Sigma-Aldrich, MO, USA) per well for 10 min at room temperature with continuous agitation on a shaker, and thereafter washed 5 times with 1 mL dH2O/well and then for 15 min with 1 mL PBS/well at room temperature with rotation. Brightfield microscopic images were captured to visualize the calcium deposits. The stained calcium deposits were eluted by incubating with 1 mL of 10% Cetylpyridinium chloride (Sigma-Aldrich, MO, USA) for 10 min at room temperature with continuous agitation. The absorbance was measured at 562 nm using a spectrophotometer (SpectraMax® L. Microplate, Molecular Devices) and the fold differentiation was calculated by dividing the absorbance for the osteogenic differentiation samples with the absorbance for the control samples (A_diff_/A_ctrl_). Cetylpyridinium chloride was used as blank.

#### 2.7.5 Karyotyping

Fetal MSCs were cultured in 75 cm^2^ tissue culture flasks as described above but with the addition of 5 ng/mL Fibroblast Growth Factor (Peprotech, NJ, USA) for 48 h before being treated with 0.4 μg/μL Colcemid (Sigma-Aldrich, MO, USA) over night, then hypotonic shock with 0.57% potassium chloride (KCl, Sigma-Aldrich, MO, USA) and fixed with methanol/acetic acid (3:1). Chromosomes from at least 20 metaphases were counted under optical microscope.

#### 2.7.6 Sterility

At the clinical site, sterility was assessed using the BactAlert system (bioMérieux, France). Briefly, 2 mL reconstitution buffer used for washing of the thawed fMSCs in the initial centrifugation was injected into a BactAlert culture bottle and incubated at 37 °C in an automated microbial detection system for 7 days.

Sterility testing by the manufacturing site was performed by Apotek Produktion & Laboratorier AB in Sweden according to European Pharmacopoeia Chapter 2.6.1. The method requires a 14-day incubation period of the test product in two different media and at two different temperatures.

#### 2.7.7 Mycoplasma

At the clinical site, mycoplasma detection was performed using the universal mycoplasma detection kit (ATCC, VA, USA). Briefly, 1 × 10^5^ fMSCs were lysed and subjected to touchdown PCR with the universal mycoplasma primers A and B according to the manufacturer's protocol. The amplicon from the test sample was analysed using 2% agarose gel electrophoresis along with a positive and negative control.

Testing of mycoplasma by the manufacturing site was performed by Minerva Analytix in Germany with PCR according to European Pharmacopoeia Chapter 2.6.14.

#### 2.7.8 Endotoxin

At the clinical site, endotoxin levels were assayed using the Endosafe® PTS™ (Charles River Laboratories, MA, USA) system consisting of LAL reagent and endotoxin controls, applied to a single use, polystyrene cartridge (Endosafe® PTS: A Portable, Kinetic Chromogenic LAL Test System, Charles River Laboratories International, Inc.; 2007). Results were automatically multiplied by the dilution factor entered into the Endosafe® system. An endotoxin value of ≤0.5 EU/mL was considered negative.

Testing of endotoxins by the manufacturing site was performed by Apotek Produktion & Laboratorier AB in Sweden according to European Pharmacopoeia Chapter 2.6.7.

## 3 Results

### 3.1 Manufacture, cryopreservation and certification of the fMSCs at the manufacturing site

Four batches of fMSCs (one research and three clinical) were successfully manufactured, labelled and cryopreserved according to established protocols at the manufacturer. Release testing was performed, and all the manufactured fMSC batches fulfilled the Product Specification as shown in Table 1. The batches were then hence certified by the manufacturer and ready for transport to the clinical site. All batched were of XX sex. No differences in cell products (XX and XY) that can be related to the sex have been identified by the manufacturer.

### 3.2 Protocol and knowledge transfer and assessment of the clinical site

The technical operators at the clinical site were previously trained and qualified in GMP-procedures, and were specifically trained in performing the first six in-house release assays described in Table 1, and also in the handling and reconstitution of the cell therapy product, first at the manufacturing site and then also at the clinical site in order to verify that the quality of the fMSCs was retained after thawing and reconstitution.

The site assessment was evaluated for suitability for dose reconstitution for the clinical trial including parameters such as quality and monitoring of cryopreservation equipment, the room used for dose reconstitution, relevant equipment and competency of operators. After review of these parameters, the clinical site was approved for handling of the fMSCs for the BOOST2B clinical trial. Lastly, a mock run imitating a real life situation was performed by the clinical site to evaluate the work flow and communication between different functions e.g. the clinicians treating the participant in the trial, ordering the fMSCs from the local GMP-facility, the technical operators thawing and reconstituting the fMSCs and transferring it to the clinical unit and simultaneously registering the time from thawing to administration of the cells to the participant, along with filling out and verifying all forms and lists as per demand in the Pharmacy Manual.

### 3.3 Transport, receival and storage of the fMSCs at the clinical site

Three separate transportations of cryopreserved fMSCs were successfully performed over 7000 km from Sweden to India. The shipment reached one of the nearest airport (Chennai International Airport or Bangalore International Airport) in India via air carriage and was then transported to the clinical site via ground transport (140 km). The time for the three transports from site to site were 56 h and 12 min, 76 h and 17 min and 178 h (7 days), respectively. The delay of the 7-day transport was due to the lock-down in India during the COVID pandemic (the transports were carried out during 2019–2021). The temperature was tracked during the full transport of the fMSCs from the manufacturing site to the clinical site and was not outside the min or max thresholds, and there were no alarms during the transport. The temperature was −193 °C, −194 °C and −194 °C at arrival respectively. The highest temperatures recorded during each transport were short increases to −182 °C, −186 °C and −188 °C respectively, which took place during landing (twice per transport). This is a well-known phenomenon previously documented by the courier.

### 3.4 Effect of long-distance transportation and handling at the clinical site on the cell viability, recovery, and short-term stability

#### 3.4.1 Effect of transportation and handling on cell viability and recovery of cryopreserved fMSCs

**Fig. 3:**
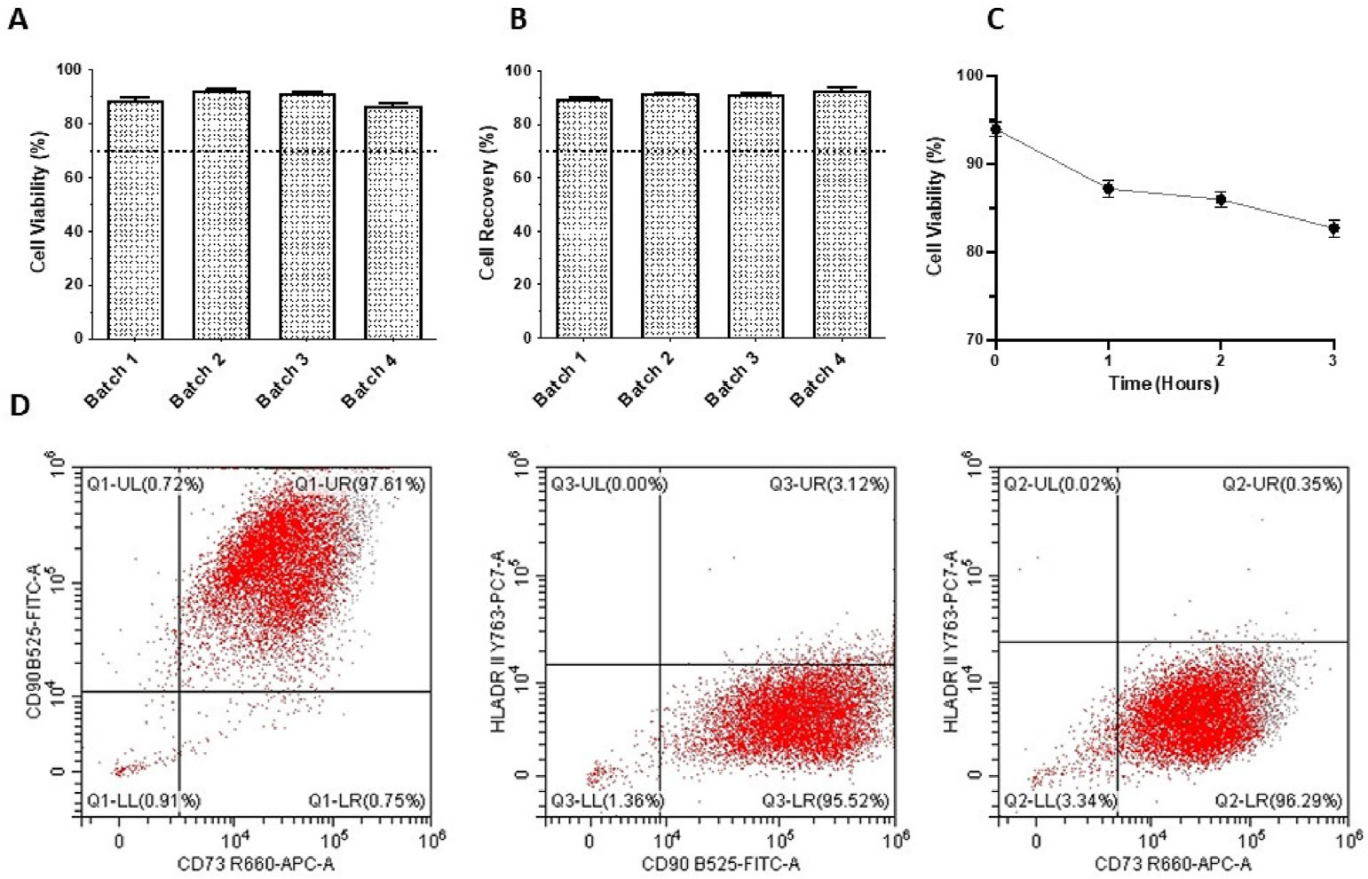
**Effect of transportation and handling on cell viability, recovery and stability over time, and surface marker expression by fMSCs. (A)** Cell viability and **(B)** cell recovery yield immediately after thawing, and **(C)** stability over time after thawing of transported cryopreserved fMSCs at the clinical site. Values are expressed as mean ± SD (n = 4). **(D)** Representative images of flowcytometry analysis of the MSC surface markers CD73 and CD90, and the non-MSC markers CD31, CD45 and HLA class II (DR) of one of the batches, which shows that 97% of the fMSCs were positive for both CD73 and CD90, and the CD73/90 positive population had less than 3% positivity for CD31, CD45 and HLA class II (DR, DP, DQ). The dotted line in A indicates the acceptance level of ≥70% viability, and in B the acceptance level of ≥70% recovery.

Prior to cryopreservation at the manufacturing site, all vials had a reported cell concentration of 5 × 10^6^ viable fMSCs/mL. The manufacturer tests the viability and recovery after cryopreservation, and the mean reported cell viability after thawing for the four batches was 92.3% (range 89.2–97.0%) and the recovery was 92.0% (range 90.0–99.6%) (Table 1). At the clinical site in India, the mean viability of the four batches tested was 90.0% (range 86.0–93.0%) and the mean recovery was 90.0% (range 88.0–92.0%) (Table 1 and Fig. 3A and B), which was not significantly different to the data obtained at the manufacturing site. The mean stability over time, i.e. the in-use stability, was at the manufacturing site determined to be 1.5 h after thawing (mean 87%, range 84–94%), and at the clinical site the mean number of viable cells was 87% (range 86–89%) at 1 h, 86% (range 85–87%) at 2 h and 82% (range 80–84%) at 3 h post reconstitution in saline and 8% HSA at room temperature with gentle intermitted rocking (Table 1 and Fig. 3C). The thawed fMSCs were plated in cell culture flasks to assess vitality. 24 h after thawing the cells had adhered and, as observed under phase contrast, displayed spindle shaped fibroblast-like appearance comparable to images from the manufacturing site (data not shown).

#### 3.4.2 Effect of transportation and handling on cell surface marker expression of cryopreserved fMSCs

The analysis shows that the transport of cryopreserved fMSCs did not significantly alter the cell surface expression of the analysed surface markers MSC and non-MSC-markers. The mean number of cells expressing CD73 and CD90 was 97.5% (range 90.9–99.6%) at the manufacturing site, and 97.9% (range 97.2–98.4%) at the clinical site (Table 1 and Fig. 3D). Surface expression of the non-MSC marker CD31 was 0.2% (range 0.0–1.0%), CD45 0.3% (range 0.0–1.1%) and HLA class II 1.2% (range 0.1–2.0%) at the manufacturing site, and corresponding data at the clinical site was 0.6% (range 0.4–0.8%), CD45 0.9% (range 0.4–2.5%) and HLA class II (DR, DP, DQ) 1.2% (range 0.2–3.1%), respectively (Table 1 and Fig. 3D).

#### 3.4.3 Effect of transportation and handling on bone differentiation of cryopreserved fMSCs

**Fig. 4:**
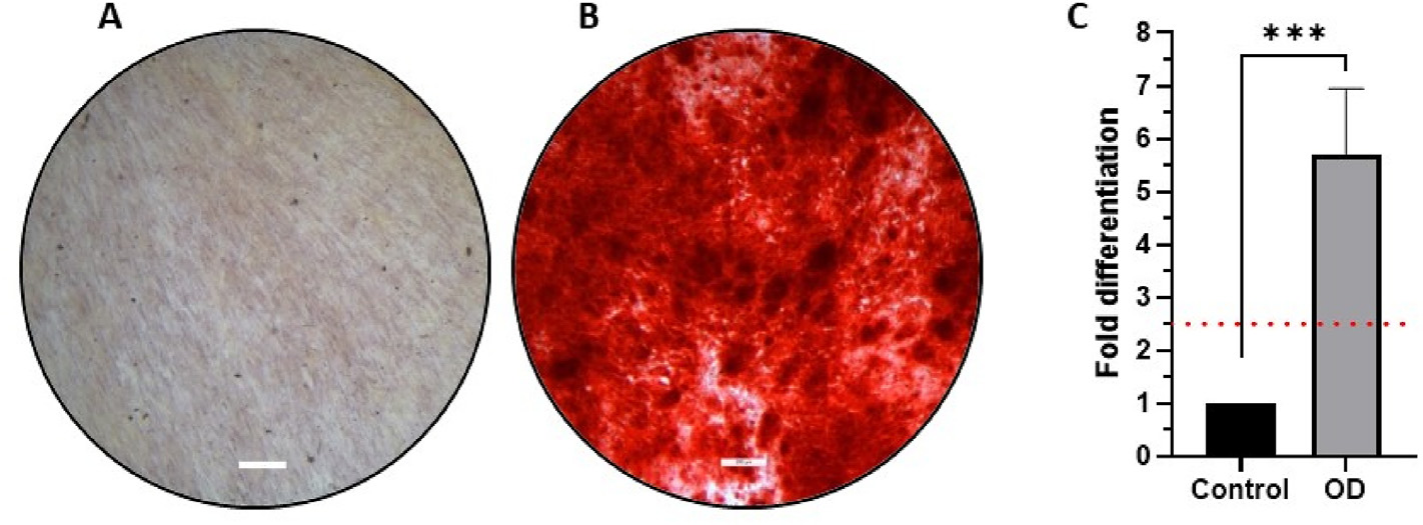
**Effect of transportation and handling on osteogenic differentiation of cryopreserved fMSCs.** Fetal MSCs were thawed, subjected to osteogenic differentiation, or kept in a fMSC culture media in vitro at the clinical site. On day 18, the cells were fixed and stained with Alizarin Red S. Typical images of a whole 12-plate well with **(A)** no osteogenic stimulation (control, normalised to 1), **(B)** osteogenic stimulation. The red stain shows extracellular mineral deposition. **(C)** Quantification of calcium deposits by elution with cetylpyridinium chloride showed a 5.7-fold increase in bone formation (OD) compared to the control samples (n = 4). Scale bar = 200 μm. OD=Osteogenic Differentiation. The red line indicates the 2.5-fold differentiation acceptance level for a positive result. ∗∗∗p < 0.001.

When stimulated to differentiate into the osteogenic lineage, all fMSCs did so, which was demonstrated with staining for calcium deposits with Alizarin Red S dye on day 18. The fMSCs kept in fMSC culture media (controls) for the same period did not differentiate into the osteogenic lineage (the background is normalised to 1 in Fig. 4C). The osteogenic stimulated fMSCs showed a mean 13.3-fold bone differentiation (range 6.3–22.5) compared to the control at the manufacturing site, versus a mean 5.7-fold bone differentiation (range 4.2–7.0) as compared to the control at the clinical site (Table 1, Fig. 4A–C, and Supplementary Figure 1), which was not statistically different. The stimulated cells showed a significant difference (p < 0.001) in eluted Alizarin Red S dye compared to the non-stimulated control cells. A 2.5-fold increase over the control is the minimum value of osteogenic differentiation, as specified in the Product Specification.

#### 3.4.4 Effect of transportation and handling on stability and sterility of cryopreserved fMSCs

All fMSCs showed a normal XX karyotype when the karyotype assay was repeated at the clinical site. Similarly, the fMSCs were sterile, negative for mycoplasma and the endotoxin levels were <0.5 EU/mL, which is considered negative.

## 4 Discussion

Many rare and orphan diseases as well as several other diseases, have until recently been considered untreatable, leaving the physicians with few alternatives to help their patients, and the patients with the option to live (or die) with ever deteriorating diseases and symptoms. ATMPs is an emerging class of drugs with the prospect of curing or treating diseases, and includes approved drugs [17], as well as cell- and gene therapy products that are currently investigated in clinical trials [2,3]. Clinical trials investigating ATMPs has proven to be challenging due to the autologous nature of the early therapies that inherently originate from the patient to be treated, leading to a sometimes complicated chain of logistics [18]. As the field moves towards development of allogeneic ATMPs, the logistics have intrinsically improved since the cells in many cases can be manufactured as an off-the-shelf product that can be thawed and used on demand [9]. In several studies, the challenges of validating bioassays widely used in the manufacturing of the same ATMP at multiple sites in multicentre studies have been discussed. Factors that need consideration is e.g. the sampling, handling and storage of the biological material as well as differences in instrumentation for read-out of the assays including Flow Cytometers and Mass spectrometers [19,20]. In all, this demonstrates the need for a wider discussion regarding method validation requirements also pointing to the added difficulty when not one method, but multiple methods and instrumentations, are to be streamlined for a consistent manufacturing with regards to quality and efficacy of the same ATMP at several manufacturing sites. A survey performed among centres Affiliated with the European Society for Blood and Marrow Transplantation, highlighted the variability in manufacturing MSCs as clinical products, and the need for harmonization between centres to be able to compare studies and improve interpretation of results [7]. A preferable approach to ensure a good quality product and comparable data would be to manufacture the cells at one site and establish functional procedures for transport, transfer of knowledge and protocols and training for handling of the product at the receiving clinical sites.

However, transferring GMP-compliant MSCs for therapeutic use across continents comes with its own challenges. The transport needs to be validated, as well as the correct handling of the cells at the receiving clinical site [12,21]. This is important in order to guarantee reproducible data from a clinical trial built on a product that in all steps of the handling retain its quality and efficacy. Therefore, in preparation for the academic clinical trial BOOST2B aiming to treat OI using fMSCs, we performed a study aiming for seamless transfer of protocols, methods and knowledge of the cell product from Sweden to India so that the highest quality of the product would be guaranteed throughout all steps [22].

We began by identifying the areas that require special attention in order to achieve the goal of a well characterized cell therapy product with retained quality when administered to the patient [6,9]. Thus, a site assessment of the receiving site was performed to confirm that the conditions in terms of staff, facilities, equipment, storage and handling was in place [12,23]. Thereafter we transferred knowledge, protocols and methods for the thawing, reconstitution and quality control of the cells to the clinical site [[24], [25], [26]]. In the first transport of cells to India, we included research vials of cells in order to control that the transportation did not affect the cells negatively, and also to practically establish and train all steps and tests, such as reconstitution, viability control, phenotype assessment, bone differentiation, karyotyping and also sterility-, mycoplasma-, and endotoxin control, to ensure that the reconstitution procedure was aseptically performed [5,9,27]. The operators in Sweden and India carried out site visits at the respective site to discuss, train and observe the handling of the cells. Furthermore, as part of the qualification process, the operators in India performed the quality tests and reconstitution steps at least three times and the cells were replated for testing of cell vitality. When handling cryopreserved products with inherent instability after reconstitution, it is advisable to perform a mock reconstitution test strictly following the Pharmacy Manual from start to finish to early identify unanticipated challenges and time constraints of for e.g. an operating theatre, and involvement of multiple clinical teams (ward, patient transfer team to radiology suite, cell reconstitution team, radiology, orthopaedic, anaesthesia). This exercise in coordination was carried out so that the stipulated in-use stability time would not be exceeded. The second and third transports to India contained the cell batches intended for clinical use that had previously been comprehensively characterized and certified by the manufacturer, and also extra vials of these cells in order to verify the identity and quality of the cell product to comply with regulations of the authorities in India.

Site assessment, transfer of protocols and methods, and technical operator qualification was successfully performed. The transferred in-house methods showed comparable levels of cell viability, cell recovery, expression of MSC/non-MSC cell surface markers, and positive bone differentiation at both sites. The *in vitro* bone differentiation assay is considered semi-quantitative, and direct numerical comparisons of differentiation levels should not be interpreted as definitive quantitative differences. Consequently, the observed 13.3-fold bone differentiation (with a range of 6.3–22.5) at the manufacturing site, in contrast to the 5.7-fold bone differentiation (ranging from 4.2 to 7.0) at the clinical site, does not unequivocally indicate dissimilar results. The threshold for a positive result is set to 2.5-fold increase over the control, and is based on historical data in combination with an extensive validation of the assay and has been confirmed by clearly visible calcium depositions when analysed in a microscope. Furthermore, sterility, endotoxin and mycoplasma results were retained after transport and reconstitution and the cells showed no discrepancies regarding the karyotype when tested at the clinical site. The methods used for sterility, endotoxin and mycoplasma differed between the sites; at the manufacturing site external laboratories performed the test according to European Pharmacopoeia, and at the clinical site approved in-house methods were used. In the European Pharmacopoeia, utilized methods must be validated according to a pre-determined schedule, and a matrix validation is required to confirm whether your specific matrix is compatible with the analytical method. In-house methods are also validated, but usually to a less stringent extent. Furthermore, the two sterility methods in this study uses different techniques; the European Pharmacopoeia method uses direct inoculation in two different media and takes 14 days, whereas the 7-day BactAlert system used at the clinical site utilise a colorimetric method to monitor the presence and production of carbon dioxide that is dissolved in the culture medium by any present microorganisms. The two methods have previously been evaluated with comparable results for the ability to detect microbial contamination [28]. For detection of any endotoxins, the standard gel-cloth method was used at the manufacturing site and the Endosafe system at the clinical site. The Endosafe system is compliant with the requirements of the European Pharmacopoeia and provide results in less than 20 min, and has been rigorously compared to the standard European Pharmacopoeia methods [29,30]. Both sites used PCR-based methods for detection of any mycoplasma. In view of testing and consistency in clinical outcomes, it is also important to match or validate all consumables at the receiving clinical site with the manufacturing site to attain reproducibility in results and to ensure that a high-quality product is administered to the trial subjects. We therefore, as far as possible, used consumables and reagents from the same manufacturer.

This study established safe transportation and efficient quality control of a cryopreserved cell therapy product to a distant site across continents for clinical application. This eliminates the requirement of a GMP facility for local production of cells as well as the need to establish and align production between manufacturing sites [7]. The study also provided practical insights into difficulties that can arise and alternative solutions to overcome the difficulties. During this study, one of the shipments experienced an extended transit time, taking 7 days to arrive rather than the anticipated 2–3 days. This incident represented the sole deviation in the study and was attributed to disruptions caused by the COVID-19 pandemic, that led to delays, redirection of the cargo, and arrival at midnight during the lockdown in India, something that could happen in also other situations, whether foreseeable or not [31,32]. Despite this setback, the delay did not compromise the integrity of the transported cell therapy products. The dry shipper's design, capable of maintaining the required temperature for up to 12 days, ensured the cells' viability, and continuous temperature monitoring was conducted throughout the shipment to guarantee the quality of the cells. In conjunction with this we emphasize the importance to use a courier that has validated their equipment, to temperature-monitor the transport, and to ship all doses for one patient collectively to avoid discontinuity in the treatment.

## 5 Conclusions

Even though this study involved only one clinical site with previous knowledge on cell therapy product we conclude that with rigours preparations, detailed planning and good communication it is possible to transport cryopreserved cells over continents and to transfer protocols and methods, in order to perform quality control of an advanced clinical cell therapy product. Moreover, we have shown that quality in both transportation and handling of the cells can be retained and that cells can be reconstituted and administered safely to patients at the clinical site. This allows for generation of uniform and high-quality data, even if the manufacturing and clinical sites are separated by long distances, which pave the way to facilitate future clinical applications in the field of cell therapies.

## Authors contributions

Conception and design of the study: LWJ, VM and CG. Acquisition of data: ÅEN, AG, SR, AK and VK. Analysis and interpretation of data and drafting and/or revising the manuscript: All authors. All authors have approved the final article.

## Funding

The BOOST2B clinical trial is an Indo-Swedish project and received funding from the Department of Biotechnology India ‒ VINNOVA (the Swedish Governmental Agency for Innovation Systems) collaboration “Indo-Swedish Innovation Call: Health and Disease Prevention 2017–2020” under the Indian grant number BT/IN/Sweden/09/VM/2017-18 and Swedish grant number 2017-00243.

## Declarations of competing interest

CG and LWJ are co-founders and co-owners of BOOST Pharma Aps. None for the other authors.
