# Supplementary material for: Successful transport across continents of GMP-manufactured and cryopreserved culture-expanded human fetal liver-derived mesenchymal stem cells for use in a clinical trial
Source: Regen Ther. 2024 Jun 24;26:324–33. doi: 10.1016/j.reth.2024.06.012 (PMC11255121; doi:10.1016/j.reth.2024.06.012)
Supplement: Multimedia component 1 [file mmc1.docx]

**Supplementary Figure 1: Phenotypic images of osteogenic differentiation of transported fMSCs.**


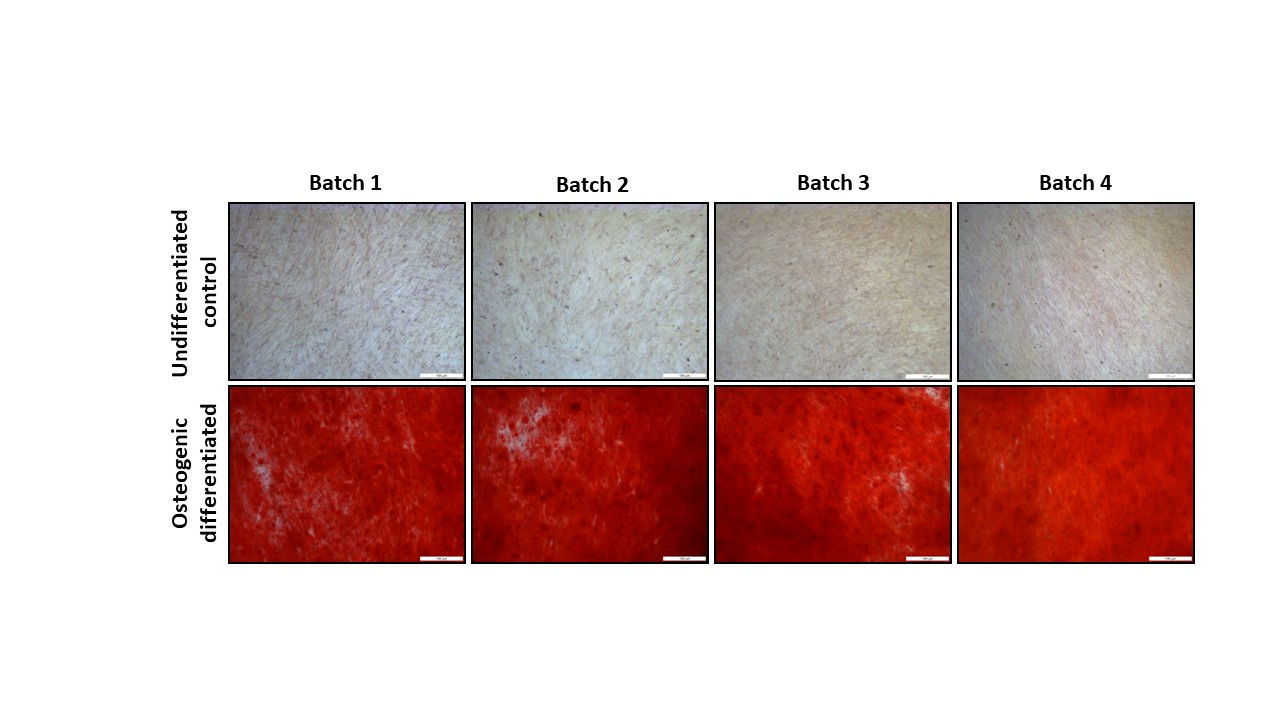


*Four batches of fetal MSCs were transported from the manufacturing site to the clinical site and stored as cryopreserved. Thereafter the cells were thawed and subjected to osteogenic differentiation or kept in fMSC culture media in vitro. On day 18, the cells were fixed and stained with Alizarin Red S and photographed using light microscopy as depicted in this figure. The red stain shows extracellular mineral deposition in osteogenically differentiated fMSCs from the four different batches. The scale bar is 100 um. The Alizarin Red dye was thereafter eluted and quantified in a spectrophotometer, please refer to Figure 4C for these results*.
